# Hypothalamic regulation of energy homeostasis: *Quo vadis*

**DOI:** 10.1007/s11154-026-10042-9

**Published:** 2026-04-29

**Authors:** Miguel López, Jeffrey M. Friedman

**Affiliations:** 1 NeurObesity Group, Department of Physiology, CIMUS, University of Santiago de Compostela, Santiago de Compostela, 15782 Spain; 2 CIBER Fisiopatología de la Obesidad y Nutrición (CIBEROBN), Santiago de Compostela, 15706 Spain; 3 Laboratory of Molecular Genetics, Howard Hughes Medical Institute, The Rockefeller University, New York, NY USA

**Keywords:** Hypothalamus, Energy homeostasis, Neuroendocrinology, Metabolism, Obesity

## Abstract

The hypothalamus orchestrates energy homeostasis via specialized neuronal populations and glial cells that integrate interoceptive signals, such as leptin, glucose, and fatty acids, through key intracellular energy sensors, like AMP-activated protein kinase (AMPK). This enables the hypothalamus to coordinate behavioral, autonomic, and neuroendocrine responses that regulate adipose mass and metabolism. This foundational Neuroendocrinology paradigm, forged through centuries of research, drives the mission of this Special Issue. From the cellular complexity of hypothalamic circuits and their hormonal regulation, to sex dimorphism and clinical relevance, as well as cutting-edge advances, such as single-cell hypothalamic mapping, circuit dynamics, and emerging therapeutic frontiers, this collection offers a comprehensive roadmap to the past, present and especially the future of hypothalamic regulation of energy homeostasis.

## *Praefatio*: Introduction

Several brain regions are involved in regulating energy balance to maintain homeostatic control of adipose tissue mass, with the hypothalamus playing a central role [1–5]. Specific cell populations in the hypothalamus respond to alterations in food availability, energy stores, and nutritional requirements, thereby eliciting counter-regulatory mechanisms with broad effects on many organ systems [1–5]. These processes involve alterations of behavior, the engagement of the autonomic nervous system [sympathetic (SNS) and parasympathetic (PSNS)], and the neuroendocrine axes. In the hypothalamus, specific groups of orexigenic and anorexigenic neurons, as well as glial cells, sense leptin and other interoceptive signals, such as glucose and fatty acids, hormones, sensory information and light/dark cues, which then project to multiple downstream sites to regulate those behavioral, autonomic, and neuroendocrine functions [1–5]. This conceptual framework, now considered fundamental to Neuroendocrinology, emerged from centuries of intense scientific inquiry [6, 7].

### *Ubi hypothalamus fuit…* Where has the hypothalamus been? The pioneers

In the 16th century, Andreas Vesalius described in *De Humani Corporis Fabrica* the first detailed anatomical representations of the pituitary stalk and the pituitary gland, located below the brain [8]. Centuries later, in the mid-19th century, Claude Bernard observed that lesions in the fourth ventricle of rabbits promoted glycosuria, demonstrating that the central nervous system could directly regulate peripheral metabolism [9]. This established the physiological foundation of the field, as well as the concept of *“fixité du milieu intérieur” (“the constancy of the internal milieu”)* [9], which would later be formalized as *“homeostasis”* by Walter Cannon [10]. In the 19th century, Auguste-Henri Forel [11] and Johann Bernhard Aloys von Gudden [12] described specific nuclei and fiber tracts at the base of the brain, which Wilhelm His, in 1893, named *“hypothalamus”* [derived from the Greek *hypó (ὑπό*,* “under”) and thálamos (θάλαµος*,* “inner chamber”* or *“couch”)*] [13]. One year later, Santiago Ramón y Cajal applied his staining techniques to generate the first detailed description of the hypothalamic neuronal architecture, revealing a complex network of cells and connections [14].

In 1901, Alfred Fröhlich described an obesity and infertility condition referred to as adiposogenital dystrophy in patients with tumors in the sella turcica, the bony depression at the skull base that houses the pituitary gland [15]. This condition, known as Fröhlich’s syndrome, was associated with the subcutaneous adiposity, hypogonadotropic hypogonadism and growth retardation. The pathophysiology of this syndrome was intensely debated at that time. While some remarkable endocrinologists, such as Harvey Cushing, proposed a pituitary origin [16, 17], Bernhard Aschner showed that hypophysectomized dogs (with an undamaged hypothalamus) did not develop obesity [18]. Three decades later Alfred W. Hetherington and Stephen W. Ranson confirmed that specific stereotaxic electrolytic lesions of the mediobasal hypothalamus that spared the pituitary gland induced obesity and the same neuroendocrine impairment described in Fröhlich’s patients [19–24]. This evidence proved that an intact hypothalamus was essential for normal endocrine function, but also for homeostatic body weight and metabolism. Further work by others, such as Ernst Scharrer and Berta Scharrer [25], Grigore Popa and Una Fielding [26, 27], George B. Wislocki and Lester S. King [28] and especially Geoffrey Harris [29, 30] then showed that blood flows from the hypothalamus (specifically from the median eminence) to the anterior pituitary gland. These advances provided the foundation for the discovery by Roger Guillemin and Andrew Schally that several different hypothalamic peptide hormone-releasing factors, such as thyrotropin-releasing hormone (TRH), gonadotropin-releasing hormone (GnRH) and somatostatin, controlled the secretion of pituitary hormones [31–35], for which they were awarded the Nobel Prize in Physiology or Medicine in 1977. Their studies established the molecular basis of the hypothalamic regulation of endocrine axes and energy homeostasis. Building upon this legacy, Wylie Vale, a key collaborator of Roger Guillemin, advanced the field further with his discovery of corticotropin-releasing hormone (CRH) and growth hormone-releasing hormone (GHRH), effectively connecting brain signals to systemic stress and growth responses [36, 37].

By that time, the parabiotic experiments with *ob/ob* and *db/db* obese mice of Douglas Coleman [38, 39] had already provided the functional evidence supporting the existence of a circulating “satiety factor” that acted on a specific receptor to regulate food intake and body weight. Parabiosis experiments performed by G. Romaine Hervey using obese rats with hypothalamic lesions further suggested that this receptor was likely to be expressed in the hypothalamus [40]. Parallel to this, Stephen C. Woods and Daniel Porte Jr. identified insulin as the first circulating signal of adiposity capable of crossing the blood-brain barrier (BBB) to act on the hypothalamus and reduce food intake [41–43], suggesting that hormones can act centrally to control of energy balance.

Two decades later, the modern era began when Jeffrey M. Friedman and colleagues discovered leptin [44], the hormone predicted by Coleman’s parabiosis experiments [38, 39]. Leptin, an adipocyte hormone, was demonstrated to play a key role in the regulation of energy homeostasis [45–49]. Consistent with Hervey’s experiments, Friedman and colleagues also demonstrated that mice with hypothalamic lesions showed a markedly increased expression of leptin in adipose tissue, suggesting that leptin acted on the hypothalamus to regulate weight [46]. This was confirmed when the group of Friedman [50], and Louis A. Tartaglia [51, 52], independently identified that the diabetes (*db*) gene encodes the leptin receptor (LEPR). These studies revealed that while the receptor is widely expressed, the signaling-competent long isoform (named Ob-Rb) was highly enriched in the hypothalamus [50–52].

Further studies by Friedman’s group, as well as Robert V. Considine and José F. Caro, showed that leptin levels are highly correlated with adipose tissue mass, establishing it as a nutritional signal that conveys information about the amount of energy stores in adipose tissue [53, 54]. These and other findings indicated that leptin was the afferent signal in a negative feedback loop regulating food intake and body weight. Rexford S. Ahima, Ronald M. Lechan and Jeffrey S. Flier [55–60], as well as Felipe F. Casanueva and Carlos Diéguez [61–64], showed that in addition to regulating energy balance, leptin regulated the neuroendocrine response to fasting. Meanwhile, Michael W. Schwartz, Denis G. Baskin, Stephen C. Woods and Daniel Porte Jr. identified the cellular targets of leptin and insulin action, including orexigenic neurons expressing neuropeptide Y (NPY), later shown to also express agouti-related peptide (AgRP), and anorexigenic neurons expressing proopiomelanocortin [POMC, the precursor of alpha-melanocyte-stimulating hormone (α-MSH)] in the arcuate nucleus of the hypothalamus (ARC) [65–70]. More recently, Jeffrey M. Friedman and colleagues identified a third leptin-responsive population in the ARC expressing basonuclin 2 (BNC2) [71].

Sadaf Farooqi and Stephen O’Rahilly translated these findings into clinical practice by showing that mutations in leptin in humans caused massive obesity that was reversed by leptin treatment [72–75]. Subsequently, Karine Clément, Philippe Froguel and Bernard Guy-Grand identified the first mutations in the human LEPR, confirming that defects in leptin signaling also lead to early-onset morbid obesity and pituitary dysfunction [76]. Julio Licinio, Christos S. Mantzoros and Philip W. Gold further expanded this clinical spectrum by describing the impact of leptin on endocrine axes and the existence of multiple hormonal defects, decreased sympathetic tone, and immune system dysfunction in adults with congenital leptin deficiency [77–81]. Of note, Mantzoros also demonstrated that leptin reversed hypothalamic amenorrhea [82, 83], providing the molecular basis for the hypothesis of Rose Frisch, who had proposed that the ability to reproduce requires a certain threshold of body fat to serve as the minimal store of energy necessary for ovulation and menstruation [84].

Giles Yeo, Sadaf Farooqi and Stephen O’Rahilly [85–88], alongside Ute Hochgeschwender [89, 90], proved that mutations in POMC, in the enzymes that process it to α-MSH, and in melanocortin 4 receptor (MC4R), identified by Roger Cone in mice [91–97], led to human obesity. Furthermore, Rubén Nogueiras, Diego Perez-Tilve and Matthias H. Tschöp demonstrated the major role of the melanocortin system in integrating peripheral signals to coordinate central and autonomic metabolic regulation [98, 99]. In separate studies, Kenji Kangawa and Masayasu Kojima discovered the stomach-derived hormone ghrelin and its hypothalamic orexigenic actions [100, 101], with Stephen R. Bloom [102–104], Felipe Casanueva and Carlos Diéguez [105–109] dissecting its hypothalamic effects on GH secretion and food intake, and Matthias H. Tschöp and Françoise Rohner-Jeanrenaud revealing its adiposity-promoting effects through the autonomic nervous system [110, 111].

### *Quid hypothalamus est…* What is the hypothalamus now?

Thus, since the beginning of the 21st century and over the following years, this framework had provided the basis for understanding how energy balance is controlled: peripheral signals, such as hormones, among them leptin, ghrelin, insulin, peptide YY (PYY), thyroid hormones, ovarian steroids, glucagon-like-peptide-1 (GLP-1) and bone morphogenetic protein 8B (BMP8B), among others, act on hypothalamic nuclei to communicate information about the energy stores, levels of nutrients in blood, and endocrine state to brain circuits. Delineation of these complex circuits, extensively mapped by Clifford B. Saper [112–115], revealed that the hypothalamus is not an isolated center but a sophisticated integrator of energy balance, sleep-wake cycles, temperature and fever, behavior and autonomic control. In this sense, the work of many scientists, such as Jeffrey M. Friedman and colleagues [71, 116–118], Michael W. Schwartz [119–121], Stephen R. Bloom [102, 122–127], Jens Brüning and C. Ronald Kahn [128–134], Serge Luquet and Richard D. Palmiter [135–141], Jeffrey S. Flier and Eleftheria Maratos-Flier [142–146], Joel Elmquist and Bradford B. Lowell [147–154], Sabrina Diano and Tamas L. Horvath [155–160], Martin G. Myers [161–163], Michael Cowley [164, 165], Lora K. Heisler [166–169], Takeshi Sakurai and Masashi Yanagisawa [170–176], Luis de Lecea and J. Gregor Sutcliffe [172, 177–179], Kamal Rahmouni [180–185], Miguel López and Antonio Vidal-Puig [186], Jens Mittag and Björn Vennström [187–189], Sebastian G. Bouret and Richard B Simerly [190–193], Rémy Burcelin and Daniel J. Drucker [194–198], Eric Fliers and Andries Kalsbeek [199, 200], along with several other groups, established the sensing of peripheral hormones by hypothalamic neurons expressing diverse neuropeptides, including POMC-derived peptides, AgRP, NPY, BNC2, melanin-concentrating hormone (MCH), and the orexins/hypocretins. These neurons, in turn, regulate whole body energy homeostasis [from feeding and energy expenditure through brown adipose tissue (BAT) thermogenesis, to liver and muscle metabolism, renal function and blood pressure, among others] via humoral and autonomic (SNS and PSNS)-mediated pathways.

However, the understanding of how this peripheral information was processed intracellularly to elicit hypothalamic responses remained unclear. Over the last quarter of a century, further advances in the field have allowed us to understand how sensing of hormones and nutrients regulates the hypothalamus and that this molecular integration is likely the basis of the precision metabolic medicine that is being built today. In 2002–2003, M. Daniel Lane revealed that mice treated with inhibitors of the lipogenic enzyme fatty acid synthase (FAS) displayed hypophagia due to the hypothalamic sensing of its substrate, malonyl-CoA [201–205]. This work, together with evidence from Tony K. Lam and Luciano Rossetti [206–211], Núria Casals, Gary D. Lopaschuk and Fausto Hegardt [212–215], as well as Céline Cruciani-Guglielmacci and Christophe Magnan [216] established that hypothalamic lipid sensing, hypothalamic fatty acid oxidation, and the activity of carnitine palmitoyltransferase-1 (CPT1) also regulated whole-body energy balance, including glucose homeostasis and hepatic glucose production. Yet the central question of how these nutrient-sensing pathways were integrated with hormonal signals remained. This led to the discovery of the hypothalamic AMP-activated protein kinase (AMPK) as a key metabolic energy sensor. The work by Caroline J. Small and David Carling [217, 218], as well as Yasuhiko Minokoshi and Barbara B. Kahn [219–226] showed that hypothalamic AMPK regulated food intake. Miguel López and colleagues revealed that, beyond its role in feeding, hypothalamic AMPK acted as a master regulator of whole‑body energy homeostasis, integrating nutrient and hormonal signals to coordinate hypothalamic fatty acid oxidation and ceramide‑induced lipotoxicity, which in turn led to the regulation of food intake, BAT thermogenesis and liver metabolism [186, 227–235]. In parallel, the work of Daniela Cota and Randy J. Seeley identified hypothalamic mechanistic target of rapamycin (mTOR) as another crucial cellular nutrient sensor [236–238]. Together, these intracellular pathways, AMPK and mTOR, as well as the maintenance of cellular proteostasis, highlighting the fundamental role of hypothalamic endoplasmic reticulum (ER) stress, as shown by Dongsheng Cai [239, 240] and Umut Ozcan [241–243], constitute the molecular “black box” that integrates peripheral cues into coordinated neuroendocrine and metabolic outputs.

Beyond signaling pathways, the maintenance of organelle integrity also emerged as a fundamental layer in the hypothalamic regulation of energy homeostasis. The work of Dongsheng Cai [244] and Rajat Singh [245, 246] showed that hypothalamic autophagy is essential for the control of food intake by modulating neuropeptide levels in response to nutrient availability. Furthermore, the pioneering research of Marc Schneeberger and Marc Claret [247–249], alongside Kamal Rahmouni [250], Sabrina Diano and Tamas L. Horvath [160, 251, 252], demonstrated that mitochondrial dynamics (the balance between fusion and fission of mitochondria) within specific hypothalamic neurons was essential for metabolic sensing.

Crucial to this integration was the link between energy balance and reproduction. Although the kisspeptin (KISS1) gene was originally identified in 1996 by Danny R. Welch as a metastasis suppressor [253], its pivotal role in the hypothalamus only emerged in 2003–2004. Nicolas de Roux [254], besides Stephanie B. Seminara, William F. Crowley Jr. ,Samuel A.J.R. Aparicio and William H. Colledge [255] discovered that mutations in KISS1 receptor, named G-protein-coupled receptor 54 (GPR54, now KISS1R), caused hypogonadotropic hypogonadism. Manuel Tena-Sempere proved how metabolic signals, such as leptin, and metabolic sensors like hypothalamic AMPK, mTOR, as well as ER stress and ceramide metabolism, precisely gate the onset of puberty and adult fertility [256–265]. Hervé Le Stunff demonstrated the role of hypothalamic ceramides in the central actions of estradiol on glucose homeostasis [266]. In this context, the work of Yong Xu and Deborah Clegg [267–269], Stephanie M. Correa and Holly A. Ingraham [270–274], Sabrina Diano and Tamas L. Horvath [157, 275, 276], Ismael González García, Cristina García-Cáceres, Manuel Tena-Sempere and Miguel López [229, 232, 235, 266, 277–280] was instrumental in demonstrating that the hypothalamus is a sexually dimorphic organ, dissecting the molecular mechanisms of estradiol action and highlighting that the biological basis of energy homeostasis cannot be fully understood without incorporating a sex perspective.

### *Quo vadis hypothalamus…* Where is the hypothalamus going?

Research over the last 10 years has also advanced our knowledge of the genetic and functional cytoarchitecture of the hypothalamus. Classically, the hypothalamus has been organized into anatomically defined neuronal clusters, called nuclei, forming interconnected neuronal circuits via axonal projections [1–5]. However, the recent developments of high-throughput single-cell and single-nucleus RNA sequencing methods have enabled the definition of cellular subpopulations with unprecedented molecular resolution, culminating in the generation of both mouse and human *HypoMaps*, by Jens Brüning and Giles Yeo [281, 282], representing a paradigm shift in our understanding of the cellular phenotypes and functions of hypothalamic neurons. Consequently, we now understand that classical neuropeptide systems are composed of molecularly distinct subpopulations with divergent functional roles; for instance, “POMC neurons” are no longer seen as a single entity, but as a mosaic of subsets with different sensing capabilities and projection targets, as demonstrated by Daniela Cota [238] and especially by Carmelo Quarta, who went further by recently identifying “Ghost” POMC neurons [283], which can reversibly adapt their functional identity in response to obesogenic stimuli, highlighting a level of hypothalamic plasticity previously unimagined. This functional diversity extends beyond the ARC to the lateral hypothalamic area (LHA). Tatiana Korotkova showed that different neuronal populations within the LHA (expressing LEPR or neurotensin, Nts) act as critical regulators of behavioral flexibility, balancing nutritional requirements with social rewards [284]. In keeping with this, Christoffer Clemmensen and Ole Kiehn recently identified a glutamatergic LHA-pedunculopontine nucleus (PPN) circuit that prioritizes safety-seeking locomotion over other essential needs, such as foraging or social interaction, by recruiting specific midbrain motor pathways [285]. The evolutionary aspects of this neuronal heterogeneity, in POMC cells, LHA neurons and other populations, such as steroidogenic factor 1 (SF1) neurons of the ventromedial nucleus of the hypothalamus (VMH), and the possible involvement of hypothalamic mechanisms regulating energy balance, for example the cell-specific roles of AMPK subunits [234, 286, 287] are questions that will require further exploration.

Modern Neuroendocrinology has transitioned from molecular and cellular identification to the precise dissection of neural circuit dynamics. Zachary A. Knight redefined our understanding of hunger and satiety, as well as body temperature [288–291]. Scott M. Sternson dissected the architecture of feeding and behavioral circuits by developing sophisticated tools such as optogenetics and CaRMA (calcium and RNA multiplexed activity) imaging [292–295]. Henning Fenselau elucidated the synaptic mechanisms and plasticity that sustain long-term energy balance [296, 297]. These advances, alongside the recent projectome-based characterization of hypothalamic neurons, such as that performed by Shengjin Xu, Hui Gong, Yan-Gang Sun and Xiaohong Xu [298], will enable a comprehensive understanding of how hypothalamic circuits orchestrate metabolic and behavioral homeostasis. Complementing this circuit-centric shift, Marc Schneeberger explores how the brain’s vasculature and immune signals coordinate with hypothalamic circuits to regulate metabolism, providing a multi-dimensional framework for understanding obesity and its associated neurodegenerative risks [299, 300].

As a result of this evidence, the hypothalamus is now understood as a complex integrated ecosystem where non-neuronal cells are active metabolic players. Astrocytes, as shown by the work of Luis M. García-Segura, Jesús Argente, and Julie A. Chowen [301–304], and more recently by Cristina García-Cáceres, Matthias H. Tschöp, and Tamas L. Horvath [305–309] modulate glucose and leptin transport, as well as synaptic activity. Microglia, as identified by Licio A. Velloso [310–312], Michael W. Schwartz [313, 314], Jesús Argente and Julie A. Chowen [315, 316] and Suneil K. Koliwad [317, 318], trigger rapid neuroinflammatory responses to high-fat diets, contributing to hypothalamic injury. Finally, tanycytes, highlighted by the work of Rubén Nogueiras, Markus Schwaninger, and Vincent Prévot [319–325], have been identified as the essential “gatekeepers” of the third ventricle, controlling the entry of peripheral hormones into the brain. Collectively, this evidence has proved an unprecedented level of structural and functional plasticity within hypothalamic neuronal and glial networks, far exceeding what was previously imagined.

Parallel to this cellular complexity, the discovery of the microbiota-gut-brain axis has added a holistic dimension to metabolic control. While the pioneering work of Nobuyuki Sudo first demonstrated that postnatal microbial colonization is essential for the normal programming of the hypothalamic stress response [326], it was not until a decade later that Gary Frost provided the first direct evidence of a gut-derived metabolite, namely acetate, that crossed the BBB to reduce appetite through a hypothalamic AMPK-inhibition [327]. Notably, recent data from Patrice D. Cani, Rubén Nogueiras, and Marc Claret have closed the circle, by describing that hypothalamic AgRP and POMC neurons are able to influence gut microbiota via neuronal and synaptic pathways in the duodenum and through increased sympathetic tone [328]. This strengthens the idea of the hypothalamus as an overall integrator of energy homeostasis, extending its reach even to the microbiota level.

Finally, this cellular and molecular complexity sets the stage for the next frontier in the field in which precision metabolic medicine will allow us to target hypothalamic mechanisms with a degree of specificity that could not have been imagined. This is exemplified by recent work by Miguel López and colleagues using small extracellular vesicles (sEVs) to target brain AMPK, potentially offering novel therapeutic avenues not only for obesity but also for its severe comorbidities, such as ischemic stroke [329–331]. Such advances transition the field from fundamental discovery toward transformative clinical applications, potentially applicable to many chronic disorders of energy balance, such as hyperthyroidism [228, 234, 332], rheumatoid arthritis [333, 334] and even cancer cachexia [335]. It also expands to the treatment of the metabolic side effects of different drugs, such as antipsychotics, where hypothalamic AMPK is involved, as demonstrated by Silje Skrede and Johan Fernø [336, 337], Xu-Feng Huang [338–340], Junzo Kamei [340–343], Margaret K. Hahn [344, 345] and Ángela M. Valverde [346–348]. This new strategy may provide an interesting and even complementary, alternative to the impressive effects of polyagonists of gut hormones and incretins developed by Brian Finan, Christoffer Clemmensen, Carmelo Quarta, Richard D. DiMarchi, Timo D. Müller and Matthias H. Tschöp [198, 349–358], which currently are the gold standard of obesity treatment.

### Why this Special Issue?

In 1932, Harvey Cushing stated: “Here in this well concealed spot, almost to be covered with a thumbnail, lies the very main-spring of primitive existence −vegetative, emotional, reproductive− on which with more or less success, man has come to superimpose a cortex of inhibitions” [359]. Nearly half a century later, Fred Plum and Robert Van Uitert emphasized: “This bit of brain, 4 grams in weight, integrates almost all higher physiological functions” [360]. A full century after Cushing’s words, our understanding of the hypothalamus continues to expand, yet we remain convinced that we have only uncovered the tip of the iceberg. Indeed, the growing molecular, cellular, anatomical, physiological, and behavioral evidence has revealed a level of complexity that grows more intricate with every new discovery.

The objective of this Special Issue was to summarize recent developments in our understanding of how the hypothalamus regulates energy balance. Accordingly, a comprehensive scheme was designed, and an impressive group of international experts contributed. We are extremely grateful to all of them. Their work has inspired a complete generation of researchers and has established the fundamental basis of hypothalamic regulation of energy homeostasis.

## Data Availability

No datasets were generated or analysed during the current study.

## Abbreviations

AgRP: agouti-related peptide
α-MSH: alpha-melanocyte-stimulating hormone
AMPK: AMP-activated protein kinase
ARC: arcuate nucleus of the hypothalamus
BAT: brown adipose tissue
BBB: blood-brain barrier
BNC2: basonuclin 2
BMP8B: bone morphogenetic protein 8B
CPT1: carnitine palmitoyltransferase-1
CRH: corticotropin-releasing hormone
ER: endoplasmic reticulum
FAS: fatty acid synthase
GH: growth hormone
GHRH: growth hormone-releasing hormone
GLP-1: glucagon-like-peptide-1
GnRH: gonadotropin-releasing hormone
GPR54: G-protein-coupled receptor 54
KISS1R: KISS1 receptor
LEPR: leptin receptor
LHA: lateral hypothalamic area
MC4R: melanocortin 4 receptor
MCH: melanin-concentrating hormone
mTOR: mechanistic target of rapamycin
NPY: neuropeptide Y
Nts: neurotensin
Ob-Rb: long isoform of leptin receptor
POMC: proopiomelanocortin
PPN: pedunculopontine nucleus
PSNS: parasympathetic nervous system
PYY: peptide YY
SF1: steroidogenic factor 1
sEVs: small extracellular vesicles
SNS: sympathetic nervous system
TRH: thyrotropin-releasing hormone
VMH: ventromedial nucleus of the hypothalamus
